# How does national power promote the improvement of the application level of electronic medical record

**DOI:** 10.3389/fpubh.2026.1830370

**Published:** 2026-06-22

**Authors:** Lang Shu, Dan Fan, Xinyue Wang, Jiangrong Luo

**Affiliations:** 1 Department of Medical Information Center, Sichuan Provincial People's Hospital, University of Electronic Science and Technology of China, Chengdu, China; 2 Department of Anesthesiology, Sichuan Provincial People's Hospital, University of Electronic Science and Technology of China, Chengdu, China

**Keywords:** electronic medical record, limited funds, low- and middle-income countries, medical digital reform, national administrative orders

## Abstract

**Background:**

Like many low- and middle-income countries, the development of electronic medical record (EMR) systems in China was slow due to the lack of funds. It was not until 2019, when the government incorporated the evaluation of EMR systerms into the assessment indicators for large public hospitals that the application of EMR systems achieved a qualitative improvement. Our hospital was one of the pilot hospitals for this reform.

**Methods:**

This study employs single-case exploratory study. China's EMR application levels are classified from 0 to 8, with Levels 1–3 representing basic electronic data collection, Levels 4–5 indicating hospital-wide integration, and Levels 6–8 reflecting regional information sharing. We tracked EMR level progression at our hospital from 2011 to 2024 and compared these trends with national and provincial averages using data from the National Health Commission's annual evaluations (2011–2024).

**Results:**

From 2011 to 2017, our hospital promoted the development of the EMR system from level 0–4 through internal demands. After 2019, the national power prompted our hospital to raise the EMR system to level 5 in 2020 and level 6 in 2023. In 2012, 2020, and 2023, the average level of EMR systems in hospitals participating in the “National Examination” across the country was 1, 2.43, and 3.24; The number of hospitals with EMR system ratings at and above level 5 was 5–7, 176, and 395.

**Conclusions:**

In countries that cannot provide sufficient funds for the reform of medical informatization, a system for rating EMR and hospital assessment similar to the one in this study can be established.

## Graphical Abstract

**Figure:**
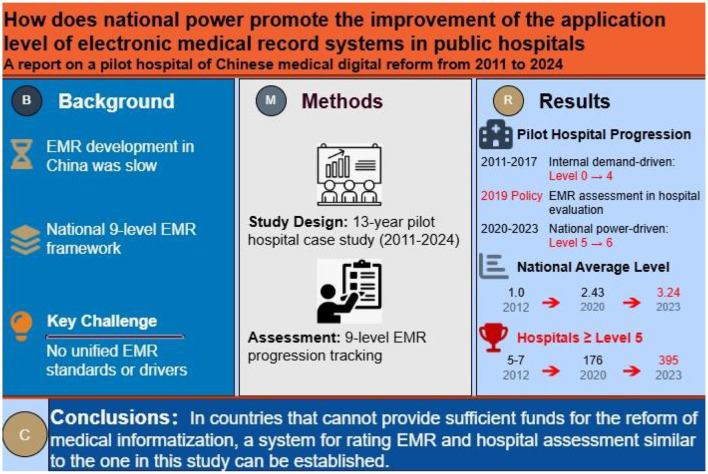

## 1 Introduction

The use of paper-based patient record systems has numerous drawbacks, such as being prone to loss, duplication, theft, fire damage, and being restricted for research purposes. During the process of handling the records, the novel coronavirus (SARS-CoV-2) and other infectious diseases may be transmitted. After the outbreak of the COVID-19 pandemic, this issue received much more attention. In the early 1960s, the United States was the first to apply computers to hospital management and established several computerized hospital management systems. Nowadays, the electronic medical record (EMR) system is one of the main systems in Hospital Management Information Systems, and its application has become quite widespread in developed countries. Research on computerized hospital management systems in China began in the late 1970s (1). For instance, from 1979 to 1986, the Heilongjiang Provincial Hospital established a multi-functional computerized hospital management system, which covered aspects such as medical record management, personnel file management, treatment quality assessment, medical statistics, pharmacy management and medical equipment management (2).

Like many developing countries, the development of EMR systems in China has been slow, due to the lack of financial support. China has a vast territory and a large population, and its economic output ranks among the top in the world. However, the per capita gross domestic product (GDP) is low and the country's investment in healthcare is insufficient. The National Bureau of Statistics of China reported that as of 2017, the total population of China was approximately 1.4 billion, accounting for about 20% of the global population. On May 19, 2020, the World Bank released the per capita GDP calculated based on the latest (2017 round) International Comparison Program. The per capita GDP of China was $14,150, which was equivalent to 23.6% of the per capita GDP of the United States in the same year. Chinese public health investment accounts for less than 8% of the GDP (3). In order to encourage public hospitals to allocate more of their limited funds to the development of EMR systems, the National Health Commission of China included the rating of the application level of EMR systems in the performance assessment of large public hospitals in 2019 (tertiary public hospitals must participate, and this is mandatory as stipulated by national administrative orders).

Sichuan Provincial People's Hospital (our hospital) is one of the pilot hospitals for this assessment. Sichuan Province covers an area of approximately 486,000 square kilometers and is the fifth largest province in China. As of 2024, its permanent resident population is 83.64 million. Our hospital is one of the large hospitals in Sichuan Province established in 1941. As of 2024, it has 4,500 beds, over 7,000 staff members, about 1,700 doctors, approximately 1.5 million outpatient visits per year, about 47,000 inpatient visits per year, and 25,000 surgeries per year. To serve such a large number of patients and staff, our hospital must enhance the application level of the EMR system to enhance service efficiency, ensure medical safety and provide decision-making data.

In 2003, our hospital established an information center. At the beginning of its establishment, there were about 5 to 6 staff members responsible for managing the computers and other equipment within the hospital. These devices were mainly used for non-medical functions such as hospital financial management. From 2011 to 2017, our hospital promoted the development of the EMR system from level 0 to 4 through internal demands (without any mandatory national administrative orders). These demands included improving service efficiency, ensuring medical safety, and providing decision-making data. After our hospital's EMR reached level 4, we have achieved information sharing across the entire hospital and no longer have the motivation to upgrade the level. Since 2011, the National Health Commission of China has organized a national evaluation of the application level of EMR systems every year (the hospitals voluntarily participated, without any mandatory requirement from the national authorities) (4). However, that was only an assessment of the single information system aspect, and it was not included in the overall assessment system of hospitals by the state.

This study reports on how our hospital has improved the application level of the EMR system from level 0 in 2011 to level 6 in 2024, with a focus on 2019 to 2024 (tertiary public hospitals must participate, and this is mandatory as stipulated by national administrative orders from 2019), during which time the level increased from 5 to 6. This research provides a demonstration for developing countries lacking financial support on how to utilize national power (the mandatory measures of national policies) to promote medical informatization (Table 1).

**Table 1:** Statement of significance.

Problem or issue:	Like many low- and middle-income countries, the development of electronic medical record (EMR) systems in China was slow due to the lack of funds. It was not until 2019, when the government incorporated the evaluation of EMR systems into the assessment indicators (national policy) for large public hospitals that the application of EMR systems achieved a qualitative improvement.
What is already known:	The United States has a clear and mature grading evaluation system (Healthcare Information and Management Systems Society Electronic Medical Record Adoption Model, HIMSS EMRAM) for EMR systems developed by the American Health Information Management Society (HIMSS), it is an international, voluntary rating and certification program. It requires payment from the hospital.
What this paper adds:	Providing an administrative review-driven model in China. It is a project that does not require payment from the hospital. The government mandates that hospitals participate in the evaluation and provides funding.
Who would benefit from the new knowledge in this paper:	In countries that cannot provide sufficient funds for the reform of medical informatization, a system for rating EMR and hospital assessment similar to the one in this study can be established.

## 2 Methods

### 2.1 Study design and case selection

This study employs a single instrumental case study design, selected because Sichuan Provincial People's Hospital is a pilot institution for China's “National Examination” policy. The hospital's characteristics (4,500 beds, >7,000 staff, ~1.5 million annual outpatient visits) make it representative of large tertiary hospitals in developing regions. Data span 2011–2024, capturing both pre-policy (2011–2018) and post-policy (2019–2024) periods.

### 2.2 Data sources

We obtained EMR level data from three sources: National-level data: Annual EMR evaluation results published by the National Health Commission's Hospital Management Institute (https://www.niha.org.cn/, 2011–2024); Provincial-level data: Sichuan Province EMR evaluation results (2011–2024); Hospital-level data: Our hospital's internal EMR implementation records, including staffing and system upgrade timelines.

### 2.3 Measurement: definition of the “national examination” and EMR application levels

The “Performance Evaluation of China's Tertiary Public Hospitals”—called the “National Examination”—started in 2019. It assesses all tertiary hospitals in China, with approximately 2,000 hospitals (the number changes slightly each year) annually, evaluating management, service quality, and efficiency. Results directly affect hospital ratings, policy support, and resource allocation (5).

**Figure 1:**
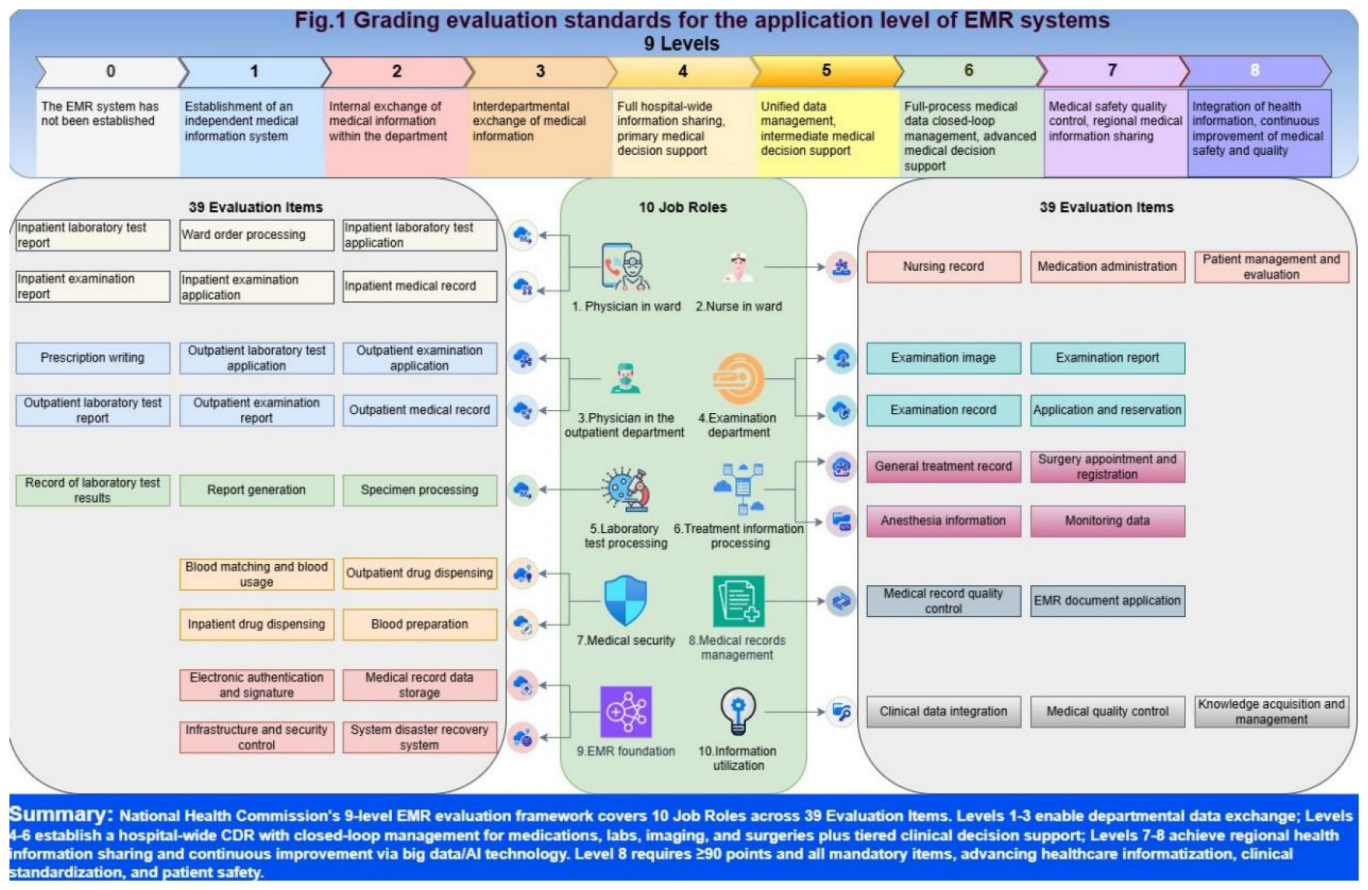
Grading evolution standards for the application level of electronic medical record systems.

EMR application level is the sole IT indicator in the National Examination (5). The grading standard has 9 levels (0–8), covering 10 job roles and 39 evaluation items (Figure 1). Released in 2011 and incorporated into the National Examination in 2019 (6). Levels 1–3: beginner (electronic data collection, intra-department sharing). Levels 4–5: intermediate (hospital-wide integration, unified data management). Levels 6–8: advanced (regional information sharing, quality control).

The selection of 39 evaluation items takes into account aspects such as medical, medical technology, medical insurance, the foundation of EMR, and information utilization, and is examined from three dimensions: the functions of the system, the effective application scope of the system, and data quality. According to the requirements corresponding to the EMR application level of 0–8 grades, the functional requirements and evaluation contents for each evaluation item were determined. The total score of the evaluation is the sum of the scores of each item during the local evaluation, and it is a quantitative indicator reflecting the overall application situation of EMR in medical institutions. The total score should not be lower than the minimum total score standard required for that level. For example, if the EMR system of a medical institution is to be evaluated at the 3rd level, the total score must not be less than 85 points. Table 2 shows the thresholds for EMR Application Levels.

**Table 2:** Thresholds for EMR application levels.

Level	Basic items	Optional items	Minimum total score (points)
8	22	4/17	220
7	22	4/17	190
6	21	15/18	170
5	20	16/19	140
4	16	10/23	110
3	14	12/25	85
2	10	15/27	55
1	5	20/32	28
0	N/A	N/A	N/A

The basic items refer to requiring specific services be in place to meet this level, while the optional items refer to the items that can be selectively met at that level. Among the optional items, “4/17” indicates that at least 4 out of the 17 optional items need to be met. Level 1 and 2 denominators only add to 37, not 39.

N/A indicates that there are no requirements.

Table 3 shows an example of the specific scoring requirements of a basic item (Ward order processing) based on a job role (Physician in ward). Due to the large amount of text in the specific scoring requirements of all basic items based on all job roles, they can be obtained from the official website of the National Health Commission at https://www.nhc.gov.cn/wjw/c100175/201812/7d64363a20cd4ea798f8343842b28d0c.shtml.

**Table 3:** An example of the specific scoring requirements of a basic item (ward order processing) based on a job role (physician in ward).

Evaluation content	Score (points)
The physician issues medical orders manually.	0
(1) Issue medical orders on the computer and record them locally. (2) Exchange data with other computers *via* disks, files.	1
The medical orders are transmitted *via* the network between the different procedures and then delivered to the nurses in the ward.	2
(1) The medical orders are available simultaneously for nurses, pharmacists and other staff to use *via* the network. (2) It is possible to obtain the availability of drugs in the pharmacy department. (3) There is a unified medical order item dictionary for the entire hospital. (4) When the medical orders are issued, it is possible to obtain the drug formulation, dosage, or at least one type of item from the examination and inspection list that is verified and prompted according to the rules of the dictionary.	3
(1)The information such as drugs, tests and examinations in the medical orders can be transmitted to the corresponding executing departments. (2)When the medical orders are issued, the related items can obtain drug knowledge, such as having the function of querying drug instructions.	4
(1)Medical orders can be uniformly managed and displayed in the hospital. (2)There is a control mechanism for the authority of physicians to issue drug treatment orders, and it supports the classification of antibacterial drugs usage management. (3)Based on the diagnosis, the situation of infectious diseases can be determined, and it can be reported to the medical administration department through the system.	5
(1) There is a reporting and handling function for adverse reactions of the prescribed drugs for medical treatment orders. (2) The prescribing physician can receive the evaluation results of their own prescriptions. (3) When issuing medical orders, it is possible to conduct automatic checks by referring to at least 4 pieces of content from the knowledge base such as drugs, examinations, tests, drug allergies, diagnoses, gender, etc., and provide prompts. (4) It is possible to monitor the status of each link of the medical order execution in real time. (5) It supports electronic application and process tracking for in-hospital consultations.	6
(1) When giving medical orders, it can automatically compare the execution and variation situations based on the clinical pathway (guidelines) requirements and the patient's specific data, prompt the input of the variation reasons and record them. (2) Based on the test results, medication use, etc., it automatically issues early warnings and provides prompts for infectious diseases, hospital infection outbreaks, etc., and supports the supplementation of information and reporting to the medical administration department for confirmed infectious diseases, hospital infection outbreaks, etc. (3)When giving medical orders, all medical records within the institution and relevant medical records from external medical institutions of the patient can be queried. (4) Automatically conduct a verification of medical orders based on the past diagnosis and treatment situations within and outside the medical institution, and provide prompts. (5)Based on the medical orders, execution status and knowledge base, automatically determine adverse event situations and provide prompts. (6) Support physicians to browse medical order records outside the hospital.	7
It can share patients‘ medical and health information and enable centralized display, including medical information within and outside the institution, health records, physical examination results, follow-up information, patients' self-collected health records (such as health records, wearable device data), etc.	8

Overall, Figure 1, Tables 2 and 3 provide detailed and clear visual representations of the differences between various levels.

### 2.4 Policy intervention: steps of countries and hospitals achieving the predetermined level of EMR

The Chinese National Health Commission has established the grading evaluation standards to promote hospitals integrate and upgrade their internal systems. Each hospital uploads system data to the national data platform. Subsequently, The National Health Commission organizes expert groups to conduct online and on-site evaluations of the hospitals, and the evaluation results are made public to allow patients to understand the hospital's level.

The results of the EMR grading evaluation will eventually be included in the “National Examination” results, and it will have the following four impacts on hospitals:

First, financial subsidies: Direct grants from the national and local governments will be given to hospitals with better assessment results. Take the “Strong Medical System Project” subsidy fund management measures issued by Shanxi Province as an example (6). The regulations therein clearly state: If a tertiary general hospital ranks among the top 100 in the “National Examination”, it will receive a provincial fiscal reward over 3 years. This sum of money can be directly used for the hospital's medical expenses. If a hospital successfully builds a national regional medical center, it will be rewarded 10 million yuan; if it successfully builds a national clinical key specialty, for each specialty, in addition to the national subsidy, the provincial government will provide an additional 3 million yuan.

Second, medical insurance payment: For Chinese patients, the medical expenses are mainly covered by the national medical insurance fund (managed by the National Healthcare Security Administration) and the patients' own payments. The main income of hospitals also comes from this, among which the national medical insurance fund is the largest “payer”. China is fully implementing new payment methods such as DRG (Diagnosis-Related Groups) payment. In simple terms, when treating pneumonia, the medical insurance pays the hospital a fixed “package price” based on the diagnosis. If the hospital achieves cost control through meticulous management and the actual cost is lower than this package price, the surplus will belong to the hospital; if the cost exceeds the limit, the hospital will bear the additional expenses itself. The allocation of the medical insurance fund's budget and policy adjustments will take into account the “National Examination” assessment results.

Third, total performance-based salary: The assessment results determine the total amount of bonuses that the hospital can offer to its employees. Higher scores mean that healthcare workers can receive higher salaries.

Fourth, appointment and dismissal of hospital directors, as well as rewards and punishments: The assessment results directly affect the position of the hospital director and are an important reference for selection, appointment, rewards and punishments (7).

If the hospital fails to pass the “National Examination”, in order to reflect the “public welfare” nature of the public hospital reform, the government's approach is not simply “punishment”, but rather “targeted breakthroughs and precise assistance” (8), helping the lagging hospitals fill in their shortcomings. The government will organize a team composed of experts in administrative management, medical management, financial management, and medical record management, to conduct on-site guidance in the hospitals. The experts will use methods such as on-site inspections and one-on-one discussions to help the hospitals identify the root causes of the problems. After the guidance, the expert team will establish a list of the hospitals' shortcomings and deficiencies, and provide feedback on each performance monitoring indicator, clearly pointing out the gaps and giving clear improvement paths and timeframes, helping the hospitals clearly define the direction for improvement.

Taking our hospital as an example, here are the steps for upgrading our EMR from level 5–6.

#### 2.4.1 Step 1: preparatory work

Through on-site investigation, we aimed to understand the functions of the mainstream EMR systems in China and the feedback on their usage. The investigation covered aspects such as the basic situation of the hospital, the number of outpatient and inpatient visits, the usage of rational drug use software, the level of application of electronic medical records, and usage suggestions.

#### 2.4.2 Step 2: establish the project implementation team

Form a project implementation team consisting of the hospital president, information center engineers, software manufacturer engineers, doctors, nurses, and technicians. The hospital president is responsible for controlling the entire project implementation process; the information center engineers are responsible for the overall construction of software and hardware, system operation and maintenance, and information security; the software manufacturer engineers are responsible for the development and implementation of each module of the software, as well as the design of interface technical solutions; doctors, nurses, and technicians are responsible for coordinating all parties and communicating with engineers to design appropriate software processes.

#### 2.4.3 Step 3: project implementation (taking rational drug use in outpatient departments as an example)

Pharmacists and IT engineers compare Level 6 standards, map drug-related functions, and analyze three prescription phases (pre-, during, post-). We will explain in Step 4 which aspects the hospital needs to improve in and what measures have been taken to upgrade from level 5–6.

#### 2.4.4 Step 4: closed-loop system construction (taking rational drug use in outpatient departments as an example)

(1) Pre-prescription

Create a hospital-wide drug knowledge base. When physicians prescribe, the system checks diagnosis, allergies, past results, and alerts on problems. From Level 5–6, three upgrades are needed: First, move from pulling allergy/medication history to recommending personalized drugs (genetics, metabolism, organ function); Second, move from real-time alerts to offering 2-3 prioritized treatment options; Third, move from rule-based blocking (>90% of high-risk errors) to combining rule results with diagnostic data for better accuracy.

Action: The president redirected landscaping funds to buy a Clinical Decision Support System and secured free staff training.

(2) During prescription

After saving, the prescription enters a review module. Pharmacists can reject (with edits or forced return) or approve. From Level 5–6, two upgrades are needed:First, automatic medication guidance sent to patient's phone; Second, online pharmacist-physician chat for real-time plan optimization.

Action: Level 5 reduced pharmacy staff by 20 and canceled annual hiring (saving ~300,000 RMB per person per year). Savings funded the Level 6 upgrade.

(3) Post-prescription

Print QR codes for personalized medication education. Random post-prescription audits trigger pop-up alerts to physicians, who can appeal. Feedback loops back to pharmacists. From Level 5–6, one upgrade is needed: real-time monitoring (e.g., antibiotic use intensity) with automatic warnings if limits exceed.

Action: China's zero-markup drug policy means hospitals bear storage and management costs. Real-time monitoring cuts operating costs, giving management strong incentive to upgrade.

## 3 Results

### 3.1 Primary finding: policy-driven acceleration

1. Pre-policy period (2011–2017): our hospital advanced from Level 0 to Level 4 over 7 years. National average EMR levels remained below 1.5 (Table 4).
2. Post-policy period (2019–2023): our hospital advanced from Level 4 to Level 6 in 4 years. National average EMR levels more than doubled (1.5 → 3.24).
3. High-performance growth: hospitals at Level 5 or above increased from 5–7 (2012) to 395 (2023)—a 55- to 79-fold increase.

**Table 4:** The years when our hospital's EMR system reached each level, as well as the comparisons with the levels of Sichuan Province and the national average (2011–2024).

Level	Content	The year when our hospital met the standards	The average level of all participating hospitals across the country	The number of hospitals at level 5 and above across the country	The average grade of the participating hospitals in Sichuan Province	The number of hospitals at level 5 and above in Sichuan Province
0	The EMR system has not been established	2011	N/A	N/A	N/A	N/A
1	Establishment of an independent medical information system	2012	1	5–7^#^	0.8	N/A
2	Internal exchange of medical information within the department	2013	1.3	7–10^#^	1	N/A
3	Interdepartmental exchange of medical information	2015	1.6	15–20^#^	1.2	N/A
4	Full hospital-wide information sharing, primary medical decision support	2017	1.5	58	1.1	N/A
5	Unified data management, intermediate medical decision support	2020	2.43	176	2	3
6	Full-process medical data closed-loop management, advanced medical decision support	2023	3.24	395	2.87	6
7	Medical safety quality control, regional medical information sharing	not qualified	N/A	4	N/A	0
8	Integration of health information, continuous improvement of medical safety and quality	not qualified	N/A	1	N/A	0

The data is sourced from the official website (https://www.niha.org.cn/) of the Hospital Management Institute of the National Health Commission, based on the annual national evaluation results announcements over the years. The results for each year are all published on the same website, but the specific URLs are slightly different. Therefore, we will not list them here.

#These are estimates since the country did not include the levels in the “National Examination”, and there was no requirement for hospitals to report it.

N/A indicates that the absence of relevant data or non-participation in the evaluation.

### 3.2 Secondary findings

**Figure 2:**
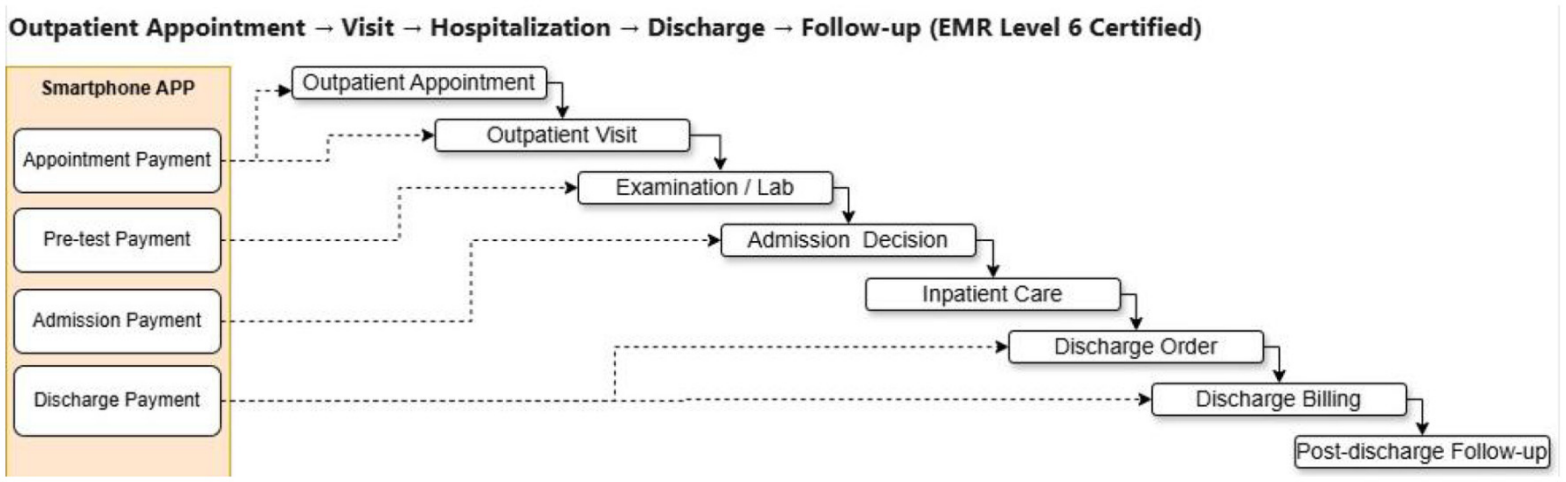
Closed-loop digital care journey.

1. Staffing solutions: our information center grew from 20 staff (2018) to 92 total personnel (2024). Notably, only 37 are government-funded; 55 are voluntarily dispatched and paid by hardware/software companies seeking business development opportunities in a real medical environment.
2. Closed-loop system achievement: by 2023, our hospital established a fully integrated EMR system covering outpatient appointments, hospitalization, discharge, and follow-up, with patient engagement *via* smartphone application (Figure 2).

## 4 Discussion

### 4.1 Interpretation of findings

This study provides empirical evidence that national administrative mandates can effectively promote EMR improvement in resource-constrained settings. Three mechanisms explain this effect:

First, incentive restructuring. Before 2019, EMR evaluation was voluntary and consequence-free. After 2019, EMR ratings became linked to four high-stakes outcomes: financial subsidies, insurance payments, staff salaries, and leadership tenure. This transformed EMR improvement from a discretionary expense to a strategic imperative.

Second, cost-shifting to private sector. The finding that 55 of 92 information center staff are company-funded (60%) demonstrates a novel public-private partnership model. Companies absorb labor costs in exchange for real-world testing environments and reputational benefits—reducing government expenditure while accelerating system development.

Third, targeted assistance for laggards. Unlike purely punitive approaches, China's “targeted breakthroughs and precise assistance” mechanism provides expert-led remediation for underperforming hospitals, preventing the widening of digital divides.

### 4.2 Analyzing from the perspective of the developing country, the reasons for implementing the evaluation of the application level of EMR systems

In 2003, China's urbanization rate was close to 40%, and it subsequently increased rapidly. By 2019, China's urbanization rate exceeded 60%, preliminarily entering the late stage of urbanization development. In 2023, China's urbanization rate reached 66.16%, marking its entry into the late stage of urbanization development (9, 10). The rapid rise in urbanization rate within a short period led to a large influx of rural populations into cities for work and settlement, resulting in an explosive growth in the number of patients in urban areas. However, as a middle- to low-income country, the increase in medical resources (such as the number of healthcare professionals) could not keep pace with the growing demand. The state had to forcefully promote informatization reforms in China's large public hospitals through institutional measures to enhance service efficiency and ensure medical safety.

Second, similar to many developing countries, China has a large population and relatively insufficient investment in healthcare. By the end of 2023, China's social medical insurance covered 1.34 billion people. Total health expenditures accounted for approximately 7% of GDP (relatively low input), with government and social medical insurance expenditures making up about 73% and personal expenditures about 27%. The average life expectancy of the population reached 78.4 years (relatively high output). These data demonstrate the success of China's social medical insurance reform (11). As the reform deepens, refined management of medical insurance funds must be strengthened, requiring medical institutions to provide authentic and detailed operational data through informatization systems.

Third, with China's own development and the increase in its interactions with the world, it has been exposed to more advanced technologies and ideas. Regardless of the country, the political commitments made by the national government to the people regarding healthcare system reforms serve as the fundamental driving force and ultimate goal for advancing such reforms. The mainstream ideology of the government is a critical factor in determining the policy agenda. The “Healthy China 2030” Planning Outline issued by the Chinese government calls for the improvement of the population health information service system (12), with specific implementation relying on the state's mandatory assessment of EMR system applications.

### 4.3 What did this governmental change do to our hospital's motivations and ability to change?

Our hospital is monitored through both national mandatory orders and financial support from the government: After 2019, the EMR rating system became a mandatory requirement for all tertiary public hospitals in China. If you don't participate, you won't be able to receive any government subsidies in the future and will face negative adjustments in health insurance payments. This is different from the HIMSS EMRAM rating system in the United States, where it is a voluntary participation for hospitals. The rating in China is free for hospitals, which is different from the HIMSS EMRAM rating in the United States, which is charged (13). Moreover, the Chinese government also provided certain subsidies to our hospital after the rating, to further enhance the EMR system (the specific details of the funds have not been disclosed).

Furthermore, although the application of the EMR system can objectively reduce the manpower costs of the hospital, the number of personnel in the information center may increase. As mentioned in the “Result” section, our hospital has learned how to deal with the situation of increasing the number of staff in the information center. Up to now, the information center of our hospital has a total of 92 staff members, among whom only 37 are paid by government funds, while 55 are voluntarily dispatched by hardware and software companies for their own business development and are paid by the companies. The dispatch of company personnel to the hospital not only helps to better maintain the system operation but also enables continuous improvement and development of new systems in a real medical environment. Moreover, the success of a company's business in a large hospital is the best advertisement for it to expand other businesses. This is a good example of government and enterprise cooperation, where the government saves funds and achieves the goal of system improvement; enterprises, encouraged by the government, see hope in the industry and promote their own business.

### 4.4 What did this governmental change do to Chinese hospital's motivations and ability to change?

The “monitoring - analysis - feedback - rectification - evaluation” closed-loop management of the Grading Evaluation of EMR reflects the country's determination to enhance the overall medical standards. It is not about eliminating anyone, but about driving all hospitals to make progress together. As mentioned in the “Result” section, it can be clearly seen that after participating in the “National Examination”, the average level of EMR in hospitals across the country and in Sichuan Province has significantly improved.

The country allocates its limited funds to the informatization construction of public hospitals. Although this initially increased the government's financial burden and reduced the burden on the hospitals' own funds, in the future, it will undoubtedly reduce the financial investment in the hospital operation process, such as reducing the cost of labor, drug management (zero markup on drugs in Chinese public hospitals), and building new public hospitals (remote medical services do not require more physical hospitals). However, to promote this process, the country needs to have strong execution power, long-term planning ability, and policy stability.

### 4.5 Limitations

Limitation 1: absence of cost data. No unified official data exist on government EMR investment across different levels. Funding is dispersed across central/local government, hospital self-funding, and social capital—preventing cost-effectiveness analysis.

Limitation 2: inability to isolate policy effects. The post-2019 period coincided with broader digitalization trends (e.g., COVID-19 telehealth expansion, smartphone proliferation). Without a control group, we cannot definitively attribute all improvement to the policy mandate. However, the sharp inflection point in 2019—after 7 years of stagnation—strongly suggests policy causality.

Limitation 3: single-country generalizability. As Odikuene et al. (14) noted for sub-Saharan Africa, EMR effectiveness depends on local healthcare systems, infrastructure, and implementation strategies (15). China's model requires strong state capacity and policy enforcement mechanisms that may be absent in other low- and middle-income countries.

Limitation 4: single-case representativeness. Our hospital is a pilot institution with above-average resources. Results may overstate what is achievable for smaller or rural hospitals.

## 5 Conclusion

For countries considering similar reforms, we recommend: First, link EMR ratings to consequential outcomes (funding, payment, accountability)—not merely publish scores. Second, establish free, government-administered evaluation to reduce hospital financial barriers. Third, provide targeted assistance to low-performing hospitals rather than punitive measures alone. Fourth, leverage public-private partnerships to offset information technology staffing costs. However, policymakers should recognize preconditions: political stability, bureaucratic capacity for annual evaluations, and healthcare system centralization sufficient to enforce mandates.

## Data Availability

The original contributions presented in the study are included in the article/supplementary material, further inquiries can be directed to the corresponding author.
